# Rapid and reliable extraction of genomic DNA from various wild-type and transgenic plants

**DOI:** 10.1186/1472-6750-4-20

**Published:** 2004-09-02

**Authors:** Tae-Jin Kang, Moon-Sik Yang

**Affiliations:** 1 Institute of Basic Science, Chonbuk National University, Jeonju 561-756, South Korea; 2 Division of Biological Sciences, Institute for Molecular Biology and Genetics, Chonbuk National University, Jeonju 561-756, South Korea

## Abstract

**Background:**

DNA extraction methods for PCR-quality DNA from calluses and plants are not time efficient, since they require that the tissues be ground in liquid nitrogen, followed by precipitation of the DNA pellet in ethanol, washing and drying the pellet, etc. The need for a rapid and simple procedure is urgent, especially when hundreds of samples need to be analyzed. Here, we describe a simple and efficient method of isolating high-quality genomic DNA for PCR amplification and enzyme digestion from calluses, various wild-type and transgenic plants.

**Results:**

We developed new rapid and reliable genomic DNA extraction method. With our developed method, plant genomic DNA extraction could be performed within 30 min. The method was as follows. Plant tissue was homogenized with salt DNA extraction buffer using hand-operated homogenizer and extracted by phenol:chloroform:isoamyl alcohol (25:24:1). After centrifugation, the supernatant was directly used for DNA template for PCR, resulting in successful amplification for RAPD from various sources of plants and specific foreign genes from transgenic plants. After precipitating the supernatant, the DNA was completely digested by restriction enzymes.

**Conclusion:**

This DNA extraction procedure promises simplicity, speed, and efficiency, both in terms of time and the amount of plant sample required. In addition, this method does not require expensive facilities for plant genomic DNA extraction.

## Background

Molecular biological studies of plants require high-quality DNA. Several DNA extraction procedures for isolating genomic DNA from various plant sources have been described, including the salt extraction method and the cetyltrimethyl ammonium bromide (CTAB) method [1] and its modifications [2,3]. The need for a rapid and simple procedure is urgent, especially when hundreds of samples need to be analyzed.

Most methods require the use of liquid nitrogen [4] or freeze-drying (lyophilization) [5,6] of tissue for the initial grinding, and these processes are unavailable in many regions of the world. After grinding the tissues in various extraction buffers, DNA is extracted with phenol-chloroform, or the extract is dialyzed against EDTA and a buffered Tris-HCl solution [7]. After extraction, the aqueous phase is concentrated, either by ethanol or isopropanol precipitation [8,9], or with microconcentrators (*e.g*., the Wizard genomic DNA purification system; Promega, USA). However, these methods are not time efficient for consistently obtaining PCR-quality DNA from calluses and plants, since they require that the tissues be ground in liquid nitrogen, followed by precipitation of the DNA pellet in ethanol, washing and drying the pellet, etc.

In our laboratory, we investigate the stability of transgenes expressed in calluses or plants transformed by nuclear or chloroplast transformation in tobacco, lettuce, potato, etc. In addition, we need high-quality genomic DNA for Southern blot analysis to confirm homologous recombination in chloroplast transformation [10]. For our purposes, we desire a simple and fast procedure for obtaining plant genomic DNA for PCR, and good-quality DNA for complete enzyme digestion for Southern blot analysis. Therefore, we present a protocol for extracting genomic DNA from fresh calluses and plant leaves that is applicable to a variety of organisms, regardless of the complexity of their genomes. In addition, we present a rapid and reliable procedure for extracting genomic DNA for PCR or Southern blot analysis from a small amount (~0.5 cm^2^) of leaf tissue.

## Results and discussion

**Figure 1:**
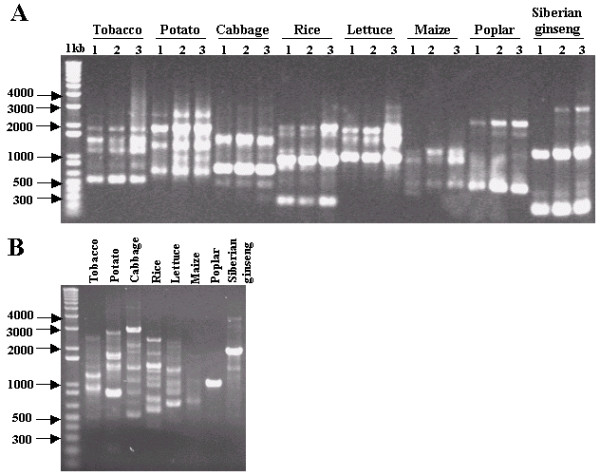
**RAPD fingerprints of all the DNA samples with primers (A) RAPD-1 (5'-CCACAGCAGT-3') and (B) RAPD-2 (5'-AAGCCCGAGG-3'). **(A) Lane 1, the DNA template was the supernatant from the first phenol:chloroform:isoamyl alcohol extraction (protocol 1); lane 2, the DNA template was the supernatant after two phenol:chloroform:isoamyl alcohol extractions and one chloroform extraction (protocol 2); lane 3, the DNA template was prepared with an additional ethanol precipitation (protocol 3). (B) PCR products amplified using only the DNA template from protocol 1. 1 kb, DNA molecular weight ladder.

We describe a simple and reproducible procedure for RAPD or PCR amplification of transgenes from various plant sources. Three different variations of the genomic DNA extraction protocol for RAPD analysis were compared. After simple plant leaf and callus tissue homogenization with DNA extraction buffer using a hand-operated homogenizer, the leaf and callus cells were lysed with 20% SDS. Then, genomic DNA was extracted with the same volume of phenol/chloroform/isoamyl alcohol (25:24:1). An aliquot of the supernatant (~5 μl) was diluted 5 fold with sterile dH_2_O, and PCR was performed using 1 μl of the diluted supernatant as a template (Figure 1, lane 1). Alternatively, after phenol/chloroform/isoamyl alcohol (25:24:1) extraction, the supernatant was transferred to a fresh tube for a second phenol/chloroform/isoamyl alcohol (25:24:1) extraction followed by a chloroform extraction. An aliquot of the supernatant (~5 μl) was diluted 5 fold with sterile dH_2_O, and PCR was performed using 1 μl of the diluted supernatant as the DNA template (Figure 1, lane 2). In the third variation, after chloroform extraction the supernatant was transferred to a fresh tube and precipitated with two volumes of ethanol. After washing the DNA pellet with 70% ethanol, the DNA pellet was dissolved in 50 μl of sterile dH_2_O containing 20 μg ml^-1 ^DNase-free RNase A. For PCR, 50 ng of the DNA were used as the template (Figure 1, lane 3).

DNA samples prepared using the three different extraction procedures (lanes 1, 2, and 3 in Figure 1) were subjected to PCR amplification using two 10-mer random primers: RAPD-1 and RAPD-2 (Genotech, Korea) (Figure 1). All the genomic DNA samples produced a clear, sharp, and reproducible PCR product when primer RAPD-1 was used for PCR amplification (Figure 1A). Although three variations of the DNA extraction procedure were used, there was little difference between lanes 1, 2, and 3. Only a difference in the intensity of the band was observed, which may be due to the different template concentrations used for the PCR reaction. This result suggests that the supernatant after the first phenol treatment (protocol 1) was sufficiently pure to be used as the DNA template for PCR amplification. Therefore, PCR amplification with another random primer, RAPD-2, was performed using the DNA template extracted using the simplest protocol (Figure 1B). The PCR amplification was successful, and the same banding pattern was seen when we repeated the PCR amplification. Therefore, we confirmed that the DNA template extracted using the simplest method was sufficient for RAPD, and it was used as the DNA template to amplify specific DNA or transgenes from transgenic calluses or plants.

**Figure 2:**
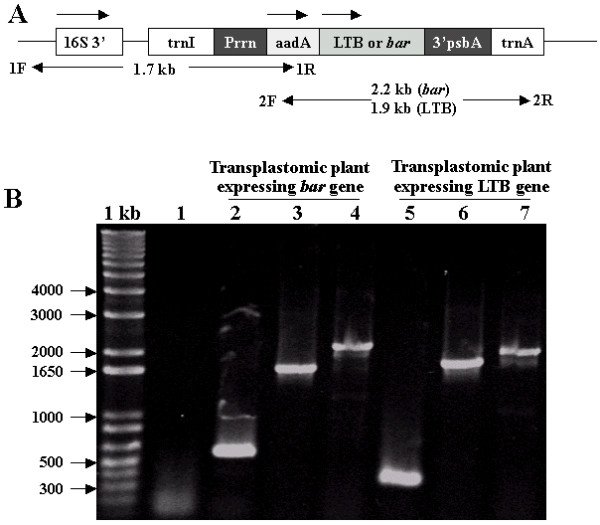
**Schematic diagram of the chloroplast genome transformed with the bar or LTB gene and PCR analysis of wild-type and chloroplast transformants. **(A) Map of the chloroplast targeting region in transplastomic plants. Arrows indicate the direction of transcription. Primer 1F is located in the native chloroplast DNA; 1R, aadA; 2F, aadA; 2R, trnA. (B) The PCR products of transplastomic plants. Lane 1, wild-type plant with primers Bar-F/Bar-R; lane 2, primers Bar-F/Bar-R produce a 550-bp fragment; lanes 3 and 6, primers 1-F/1-R produce a 1700-bp fragment; lane 4, primers 2-F/2-R produce a 2200-bp fragment containing the bar gene; lane 5, primers LTB-F/LTB-R produce a 380-bp fragment; lane 7, primers 2-F/2-R produce a 1900-bp fragment containing the LTB gene. 1 kb, DNA molecular weight ladder.

**Figure 3:**
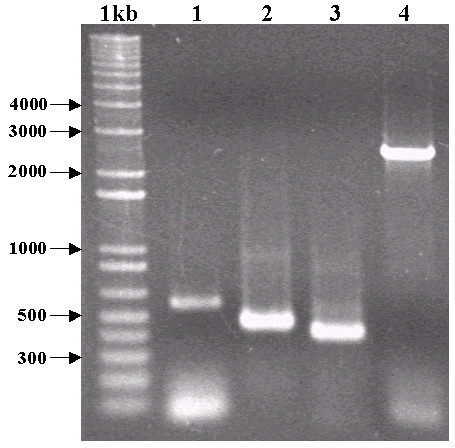
**PCR amplification products from transgenic plants and calluses. **Lane 1, α-interferon in transgenic calluses from Siberian ginseng; lane 2, Core epitope of PEDV; lane 3, LTB; lane 4, A plus B subunits of *Helicobacter pylori *urease. 1 kb, DNA molecular weight ladder.

To examine the presence of *bar *[11,12] or the LTB gene [13] at a directed site in the chloroplast DNA after homologous recombination in transplastomic tobacco plants, putative transformants were screened by PCR analysis (Figure 2). PCR amplification using primer combinations Bar-F/Bar-R, 1-F/1-R, and LTB-F/LTB-R resulted in 550-, 1700-, and 380-bp fragments, respectively. Primers 2-F/2-R produced 2200- or 1900-bp fragments containing *bar *and LTB, respectively, which confirmed the site-specific integration in the chloroplast genome (Table 1). No detectable product was produced using genomic DNA from wild-type plants (Figure 2B, lane 1), demonstrating the specificity of these primers and genomic DNA extracts. Therefore, we concluded that chloroplast DNA was also amplified, although we did not use liquid nitrogen, but simply used a hand-operated homogenizer with a plastic tip. We also successfully amplified specific foreign genes from transgenic tobacco plants transformed using the nuclear transformation method, including the α-interferon (550 bp) [14], the core epitope of the PEDV gene (420 bp) [15,16], the LTB gene (380 bp) [17], and the A plus B subunit of the *Helicobacter pylori *urease gene (2450 bp) [18] (Figure 3). Specific PCR amplification was also conducted using transgenic calluses as well as transgenic plants. In transgenic calluses derived from Siberian ginseng plants, α-interferon was successfully amplified, showing a 580-bp fragment in 1% agarose gels.

**Figure 4:**
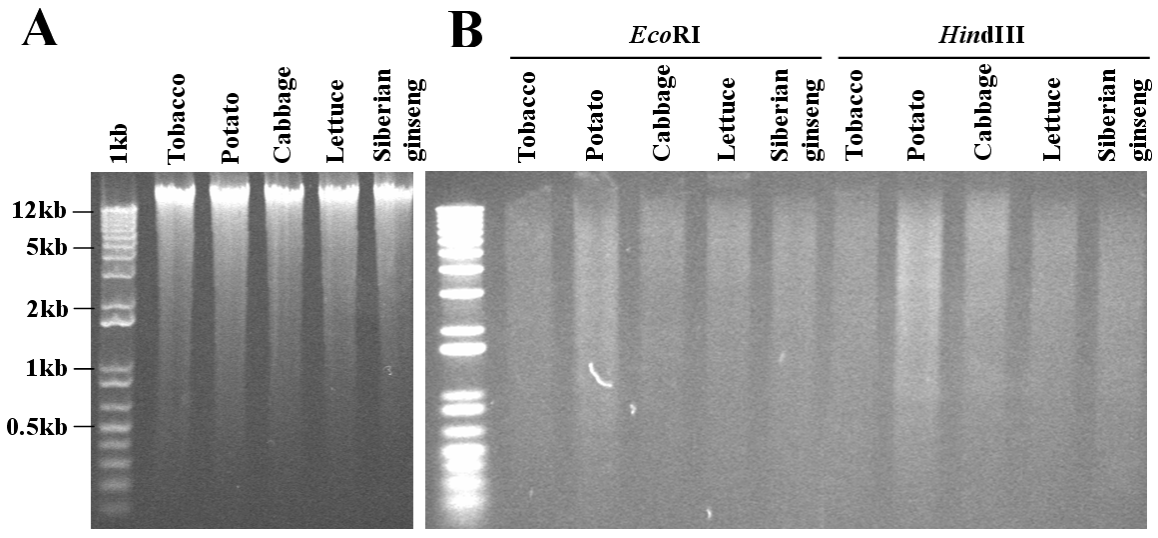
**Agarose gel electrophoresis of undigested and digested genomic DNA. **(A) Genomic DNA from five different plants with 5 μg of genomic DNA loaded from each sample. (B) Genomic DNA digested with the restriction enzymes *Eco*RI and *Hin*dIII. 1 kb, DNA molecular weight ladder.

Using the third protocol, the DNA concentrations obtained were between 20 and 30 μg/0.5 cm^2 ^plant leaf, and the absorbance ratios (A_260_/A_280_) were between 1.7 and 2.0. However, the DNA concentrations from rice, maize, and poplar were relatively low (< 3 μg). This may be because homogenization using a hand-operated homogenizer with a plastic tip is incomplete, since the leaves of these plants are stronger than the leaves of tobacco, potato, cabbage, lettuce, and Siberian ginseng. Genomic DNA from various plant sources was electrophoresed on 1% agarose gels, and high-molecular-weight DNA was obtained (Figure 4A). When the genomic DNA was digested with *Eco*RI and *Hin*dIII, the DNA was completely digested, and could be used for Southern blot analysis. Therefore, we concluded that the purity and quality of the genomic DNA was sufficient for enzyme digestion.

There are many advantages in using our genomic DNA extraction method to obtain template for PCR amplification. Many different plants could be amplified using the same DNA extraction method and the same PCR protocol. Using this protocol, we successfully amplified DNA repeatedly from all eight plant sources examined. Our procedure is not only very simple, but is also time and cost effective. After homogenization in DNA extraction buffer using a hand-operated homogenizer, the template DNA for PCR could be extracted by phenol/chloroform/isopropyl alcohol treatment. Since this method does not require liquid nitrogen, expensive commercial DNA extraction kits, or ethanol precipitation to produce DNA template for PCR, we can save considerable time and expense. The time required for our DNA extraction method is less than 30 min, which is extraordinary compared with other genomic DNA extraction methods. With our procedure, leaf tissue (~0.5 cm^2^) is put in a 1.5-ml microfuge tube and homogenized directly; consequently, a very small sample is required for DNA extraction. There is no sample waste with our method, whereas much larger samples are required when plant samples are ground in a mortar and pestle with liquid nitrogen and transferred to a tube. Previously reported techniques require several steps [19], use of expensive enzymes such as proteinase K [20], or beads and shakers [21]. Although the protocol for one-step plant DNA isolation was developed by Burr et al. [22], if plant material more than 1 mm^2 ^was used in the extraction, co-extracts (e.g., chlorophyll) were extracted alongside the DNA and inhibited the PCR. On the contrary, our protocol does not require appropriate sample size to extract DNA. Warner et al. [23] also reported a rapid DNA extraction method in barley, which requires NaOH. However, the extracted DNA samples were easily degraded. The DNA samples extracted by our protocol were very stable and could be stored for a long time without degradation.

We find the new method very useful in our laboratory, since limited transgenic plant tissue or callus is sometimes available in a culture bottle. Therefore, the simplicity, efficiency, speed, and lack of a requirement for expensive facilities make our method an attractive alternative to existing methods of genomic DNA extraction.

## Conclusions

Our objective was to extract genomic DNA with a simple and fast procedure for PCR and enzyme digestion. The present protocol is for extracting genomic DNA from fresh calluses or plant leaf tissues that is applicable to a variety of organisms, regardless of the complexity of their genomes. Our procedure is not only very simple, but is also time and cost effective. Since this method does not require liquid nitrogen, expensive commercial DNA extraction kits, or ethanol precipitation to produce DNA template for PCR, we can save considerable time and expense. In addition, a very small sample is required for DNA extraction.

## Methods

### Plant material

We examined plant material from plant collections commonly used for foreign gene expression: tobacco (*Nicotiana tabacum*), potato (*Solanum tuberosum*), cabbage (*Brassica oleracea*), rice (*Oryza sativa*), lettuce (*Lactuca sativa*), maize (*Zea mays*), poplar (*Populus nigra*), and Siberian ginseng (*Eleutherococcus senticosus*). The plants used for genomic DNA extraction were grown in a culture room or greenhouse. Tobacco, potato, cabbage, lettuce, and Siberian ginseng were grown in a culture room. Seeds were surface-sterilized with 70% ethanol for 3 min, and then with 10% sodium hypochlorite for 15 min. The seeds were washed five times in sterile water and placed in Petri dishes containing 4.6 g l^-1 ^MS salts [24], 30 g l^-1 ^sucrose, and 7.5 g l^-1 ^bactoagar at pH 5.7. The seeds were grown in a controlled environment at 25°C on a 16-h continuous light and 8-h dark daily cycle. Rice, maize, and poplar plants were grown in a greenhouse for genomic DNA extraction. Transgenic tobacco plants and Siberian ginseng calluses were also used to extract genomic DNA and to confirm foreign gene insertion by PCR amplification.

### DNA extraction (Figure 5)

We tested three different variations of the genomic DNA extraction procedure. About 0.5 cm^2 ^of culture room- or greenhouse-grown plant leaves were put in a 1.5-ml microfuge tube. The leaf tissue was homogenized in 50 μl DNA extraction buffer (500 mM NaCl, 100 mM Tris-HCl pH 7.5, and 50 mM EDTA pH 7.5), using a hand-operated homogenizer (Sigma, Z35997-1) with a plastic pestle, for 15~20 s. After an initial homogenization, another 150 μl of DNA extraction buffer were added and homogenized with the same homogenizer for 15~20 s. Then, 20 μl of 20% SDS were added and vortexed for 30 s. The samples were incubated at 65°C for 10 min for cell lysis. At this point, three different DNA extraction protocols were used for PCR amplification. Protocol 1: An equal volume of phenol/chloroform/isoamyl alcohol (25:24:1) was added to the samples, mixed by vortexing for 30 s, and then centrifuged at 10,000 g for 3 min at 4°C. The supernatant was diluted 5 fold, and 1 μl of the supernatant was used as the DNA template for PCR analysis. Protocol 2: The supernatant from protocol 1 was transferred to a fresh tube and extracted one more time with phenol/chloroform/isoamyl alcohol (25:24:1) and then with chloroform. The supernatant was diluted 5 fold, and 1 μl of the supernatant was used as the DNA template for PCR analysis. Protocol 3: The supernatant from protocol 2 was transferred to a fresh tube, and a double volume of ethanol was added to each sample, mixed well, and the samples were incubated at -20°C for 30 min. The samples were then centrifuged at 10,000 g for 10 min at 4°C. The pellet was washed with 70% ethanol, dried, and resuspended in sterile dH_2_O containing 20 μg/ml DNase-free RNase A. The concentration and purity were determined from the A_260_/A_280 _ratio using a spectrophotometer. Five micrograms of each genomic DNA sample were incubated at 37°C for 3 h for complete digestion with 20 U of *Eco*RI and *Hin*dIII (Life Technologies, USA) in a total volume of 100 μl and analyzed on 1.0% agarose gels using 15 μl aliquots of the reaction mixture.

### Analysis of DNA and PCR amplifications

Five micrograms of each genomic DNA sample measured by spectrophotometer were incubated at 37°C for 3 h for complete digestion with 20 U of *Eco*RI and *Hin*dIII in a total volume of 100 μl and analyzed on 1.0% agarose gels using 15 μl aliquots of the reaction mixture. By using the genomic DNA isolated from the leaves or calluses of wild-type and transgenic plants, PCR amplifications were performed on a Perkin Elmer GeneAmp PCR System 2400 (Biorad, USA) in a total volume of 25 μl containing 1 × PCR buffer, 0.2 mM dNTP, 10 pmol of each primer (Table 1), 50 ng template DNA from plants, and 0.25 U Ex-Taq DNA polymerase (Takara, Japan) using the following profile: a 3-min denaturation at 94°C and 40 cycles of 1-min denaturation at 94°C, 1-min annealing at 37°C for RAPD or 55°C for specific transgene amplification, and a 2-min extension at 72°C, followed by a final extension at 72°C for 7 min. The PCR products were resolved by electrophoresis in 1.0% agarose gels.

## Authors' contributions

TJK developed the method and performed majority of the experiments. MSY provided technical assistance, funding and supervision for the work. All authors have read and approved the final manuscript.

## Figures and Tables

**Figure 5:**
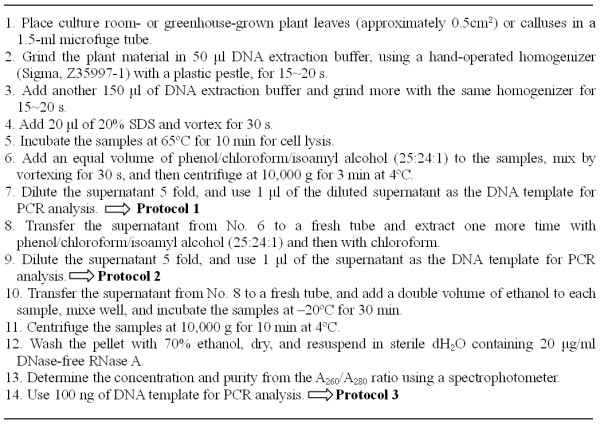
Three different DNA extraction protocols for calluses and plants

**Table 1:** Primers used in this study

**Primer Name**	**Target Amplified**	**Size of Product**	**Primers (5'-3')**
RAPD-1	Random		CCACAGCAGT
RAPD-2	Random		AAGCCCGAGG
1-F	Chloroplast	1700 bp	AAAACCCGTCCTCAGTTCGGATTGC
1-R			CCGCGTTGTTTCATCAAGCCTTACG
2-F	Chloroplast	1900 bp(LTB)	CTGTAGAAGTCACCATTGTTGTGC
2-R		2200 bp (*bar*)	TGACTGCCCACCTGAGAGCGGACA
Bar-F	*Bar*	550 bp	CGAGACAAGCACGGTCAACTTC
Bar-R			AAACCCACGTCATGCCAGTTC
LTB-F	B subunit of *E. coli *heat-labile enterotoxin	380 bp	ATGGCTCCCCAGTCTATTACAG
LTB-R			CTAGTTTTCCATACTGATTGC
PEDV-F	Porcine epidemic diarrhea virus	420 bp	TCTATGGTTACTTTGCCATC
PEDV-R			AATTAAACGTCTGTGATACC
Ure-F	A and B subunits of *Helicobacter pylori *urease	2450 bp	GCCACCATGAAACTCACCCCAAAAG
Ure-R			GGTACCCTAGAAAATGCTAAAGAGTTG
IFN-F	α-Interferon	580 bp	ATGGCCTCGCCCTTTGCTTTAC
IFN-R			CTCTTATTCCTTCCTCCTTAATC

F, forward primer; R, reverse primer
